# Reduced adaptability to balance perturbations in older adults with probable cognitive impairment after a severe fall

**DOI:** 10.1371/journal.pone.0305067

**Published:** 2024-07-10

**Authors:** Malte Voß, Tania Zieschang, Laura Schmidt, Michel Hackbarth, Jessica Koschate, Tim Stuckenschneider

**Affiliations:** Department for Health Services Research, Geriatric Medicine, School of Medicine and Health Services, Carl von Ossietzky University, Oldenburg, Lower Saxony, Germany; Kennedy Krieger Institute/Johns Hopkins University School of Medicine, UNITED STATES

## Abstract

Falls in older individuals often result from unexpected balance disturbances during walking, necessitating the analysis of recovery strategies for effective falls prevention. This becomes particularly crucial for individuals with cognitive impairment, who face a higher fall risk compared to cognitively healthy adults. Hence, our study aimed to compare the recovery response to standardized walking perturbations on a treadmill between older adults with cognitive impairment and cognitively healthy older adults. 36 individuals with a recent history of a severe fall, leading to an emergency department visit without subsequent admission, were stratified into two groups (with and without probable cognitive impairment) based on scores of the Montreal Cognitive Assessment. Recovery performance was quantified using force plate data from a perturbation treadmill (M-Gait, Motek Medical B.V., Amsterdam, the Netherlands), specifically evaluating the number of steps needed to restore step length and width to pre perturbation baseline across two trials of nine different perturbations. Individuals with cognitive impairment (n = 18, mean age: 74.7) required significantly (p = 0.045, Cohen’s d = 0.69) more steps to recover total steps after perturbations compared to cognitively healthy individuals (n = 18, mean age: 69.7). While step width recovery was similar between the groups, those with probable cognitive impairment required significantly more steps to recover their step length (p = 0.039, Cohen’s *d* = 0.72). Thus, our findings indicate that older adults with probable cognitive impairment manifest inferior gait adaptability, especially in adapting step length, potentially underscoring a critical aspect for effective falls prevention in this population.

## Introduction

Older adults with cognitive impairment (OACI) are particularly prone for falls with a two-to-three-fold higher risk than their cognitively healthy counterparts (OACH) [1]. The majority of falls occur during walking primarily induced by unanticipated disturbances in balance [2, 3], frequently stemming from slips or trips. These balance perturbations induce abrupt shifts in an individual’s center of mass, potentially resulting in falls with physical repercussions such as injuries [4, 5], as well as psychosocial consequences such as concerns of falling [6].

As task-specificity is integral to physical training, perturbation-based balance training (PBT) has become increasingly important in falls prevention efforts [7–9]. Vice versa, the measurement of an individual’s reactive dynamic balance in response to perturbations, which remains still elusive [10], may serve as an early indicator of future fall risk and a target for interventions [11], notably research in individuals with cognitive impairments is missing [12–14].

Different outcome measures such as spatiotemporal or kinetic parameters have been explored previously [10]. However, many of these methods require marker-based recordings, which are rather expensive and time consuming. Therefore, these methods may be particularly challenging to apply in clinical settings. Using the data of force plates, which are often integrated in treadmills, may present a cost- and time-efficient alternative to assess reactive dynamic balance by analyzing the time or number of steps needed to restore gait kinematics after a perturbation [15].

A previous investigation demonstrated diminished reactive balance control to stance perturbations in individuals with mild cognitive impairment compared to their cognitively healthy counterparts [16]. However, there is a lack of studies during walking. Gerards and colleagues examined the walking adaptability to perturbations in older individuals with or without a history of falls, utilizing the margin of stability to quantify the number of recovery steps. Their findings indicated that fallers exhibited inferior gait adaptability [11]. Notably, their study did not address individuals with a recent severe fall, which is defined as an incident prompting a visit to the emergency department (ED) or those with cognitive impairment. According to global guidelines for falls prevention, these groups are at an elevated risk for subsequent falls and functional decline [17].

Our objective was to evaluate the recovery performance to perturbations during walking in individuals who had experienced a severe fall requiring presentation to the ED, while accounting for their cognitive performance. This investigation aims to offer insights into the future fall risk and potential targets for interventions within this high-risk cohort for functional decline. We hypothesize that OACI will demonstrate a diminished recovery performance in response to standardized perturbations.

## Material and methods

### Participants

All participants were recruited for the SeFallED study, which is a mixed methods study ongoing at the Carl von Ossietzky University Oldenburg, Germany [18]. For the current exploratory sub-study, baseline data from participants, who underwent assessments in the gait laboratory between November 2021 and May 2023, were used. Recruitment for the SeFallED study started on November 15, 2021. The study is in accordance with the declaration of Helsinki (1974), has been prospectively registered in the German Clinical Trials Register (DRKS-ID: 00025949), and was approved by the Medical Ethics Committee of the University Oldenburg (number 2021–120). The recruitment procedures have been previously described [18, 19]. In summary, participants provided written consent for future contact while in the ED to a member of the study team. After being contacted via telephone, a trained member of the study team visited the participants at their home to procure their written informed consent for participation. In cases involving individuals with significant cognitive impairment or dementia, consent for participation was additionally obtained from their legal representative.

Eligible Participants were 60 years or older, presented to the ED of either the Klinikum or the Evangelisches Krankenhaus Oldenburg after a fall and were discharged within 72 hours [18]. Besides complete dependency on walking aids or subjectively not being able to walk 400m, other exclusion criteria were neurodegenerative diseases, a previous history of a stroke or an incomplete paraplegia. Furthermore, individuals, who hold on to the handrail throughout the whole trial, were not included in the analysis for recovery performance as this behavior may not reflect real world scenarios and may influence recovery responses.

Participants were stratified into OACH and OACI using the score of the Montreal Cognitive Assessment (MoCA) (maximum of 30 points) [20], setting the cutoff for probable cognitive impairment at ≤ 24 points to reduce false positives [21].

### Study overview

Suitable participants were addressed in the ED to receive consent for being contacted via telephone. Within a week after discharge, a member of the study team scheduled a home visit to obtain written informed consent. During this visit, an extensive geriatric assessment was conducted. Afterwards participants were invited to visit the gait laboratory. All details of the assessment battery have been published before [18].

### Reactive dynamic balance assessment

Reactive dynamic balance was assessed using the M-Gait treadmill (Motek Medical B.V., Amsterdam, the Netherlands). The participants were secured during treadmill walking using a safety harness and were instructed to not use the handrails, if possible. Due to ethical concerns, handrails were not removed from the treadmill.

**Fig 1:**
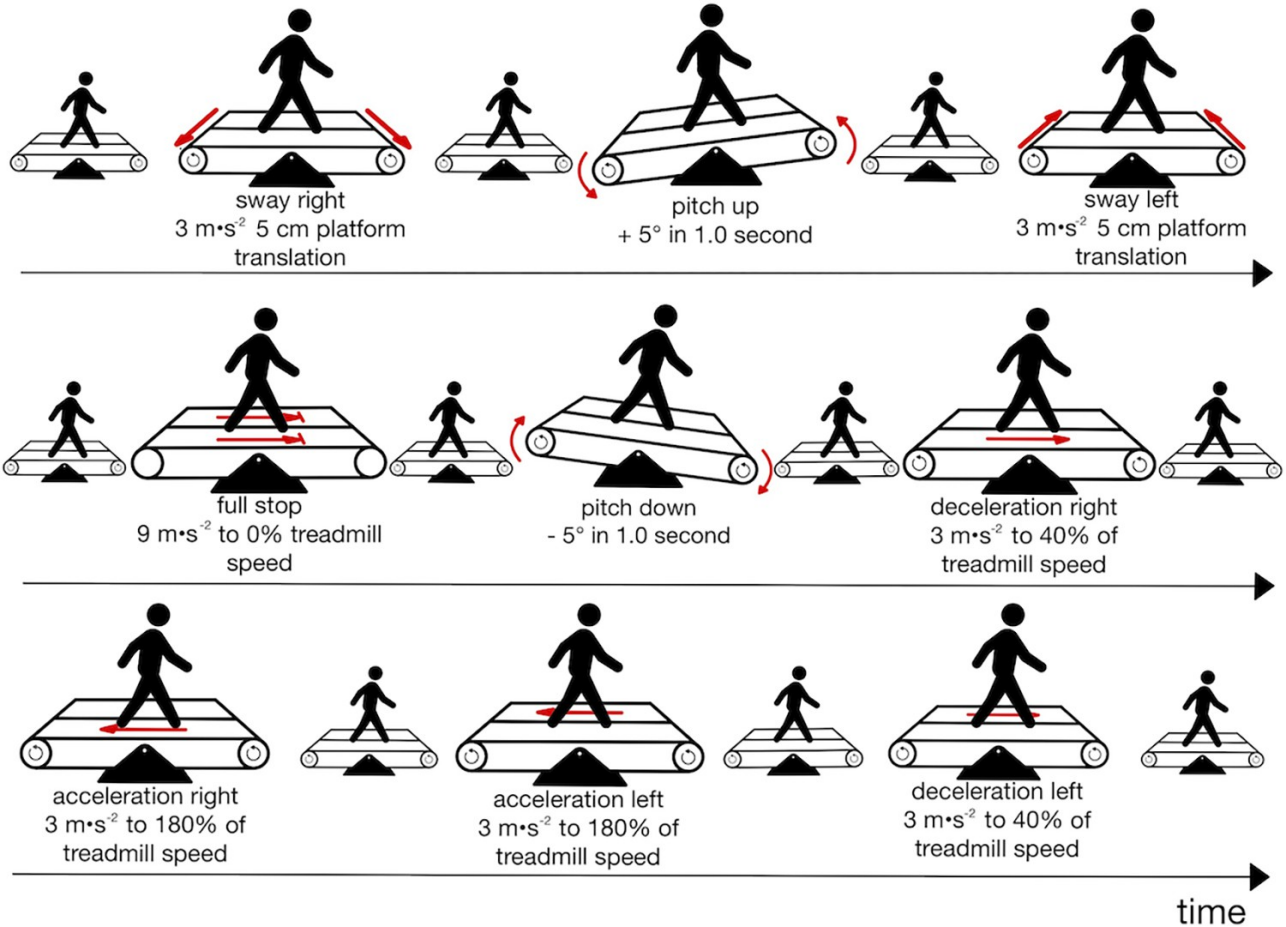
A schematic depiction of the perturbation protocol used.

After a familiarization period of six minutes, during which participants identified their individualized preferred treadmill gait speed, they underwent a perturbation protocol twice, with a two-minute break between each trial. It included nine perturbations (two slips for each leg, two trips for each leg, one emergency stop, two sways and one pitch–and—5° respectively) and a sufficient washout period of twenty to thirty seconds between the perturbations [15, 22]. Perturbations were triggered through the integrated force plates at initial foot contact through custom application as soon as the vertical force component ranged between 40 N and 200 N, and the impulse exceeded 800 Ns (D-Flow version 3.34.2, Motek Medical BV, Amsterdam, The Netherlands). Using the split belt option, each leg was perturbed in anterior-posterior direction twice, mimicking slips and trips. Slips were simulated by an acceleration of 3 m∙s^-2^ to a maximum of 180% of treadmill walking speed for 0.42 s. Trips were provoked similarly with a deceleration of 3 m∙s^-2^ to a minimum of 40% of treadmill walking speed, which is in line with previous research [23–26]. Besides these single leg perturbations, an emergency stop as in public transport was imitated with both belts decelerating by 9 m∙s^-2^ for 0.12 s [25, 27]. Further, the protocol included two contralateral sways, perturbing each leg once by a 5-cm platform translation in 3 m∙s^-2^ [25, 26]. A pitch of + and– 5 degrees respectively, with a duration of 1.0 s imitated small slope changes as present on sidewalks or at bus stops. The full protocol has been published before and a schematic illustration of the perturbations is presented in Fig 1 [18].

#### Reactive dynamic balance assessment–outcome measures

In addition to assessing overground and treadmill walking speed, outcome measures include the quantification of steps required to return to baseline levels. Consistent with previous research [11, 28], walking adaptability in this study was evaluated by examining recovery performance across the nine distinct perturbations conducted over two trials. Beyond the comprehensive analysis of total recovery, which incorporates both step width and step length, we conducted separate analyses for step width and step length recovery. The quantification of each participant’s recovery performance (total steps, step length, step width) adhered to the methodology outlined by Rosenblum and colleagues [15].

### Data curation and outcome calculations

**Fig 2:**
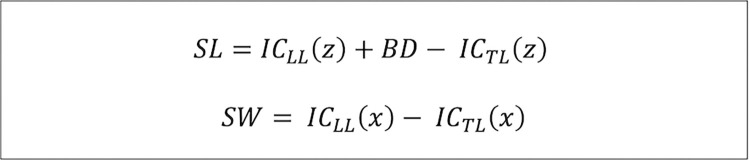
Equation used to calculate recovery performance (taken and modified from [16]); SL and SW are step length and step width, respectively, z represents anterior-posterior direction, x stands for mediolateral direction, IC_LL_ is the initial contact of the leading limb, IC_TL_ is the initial contact of the trailing limb and BD is the belt distance travelled while the leading limb is in swing phase.

Ground reaction forces were processed using a custom MATLAB (The MathWorks Inc., version 9.12.0.1956245 (R2022a), Natrick, MA, USA) script. Force, momentum, and center of pressure (CoP) data were filtered using a second order lowpass Butterworth filter with a cutoff frequency of 20 Hz in line with previous approaches [29, 30]. Following previous recommendations, the CoP data was set to zero if vertical force components were below 80 N [30]. Initial contact was defined as the local maxima in CoP measured in anteroposterior direction. Similarly, toe off was defined as the local minima. To calculate step length and step width the formula published by Rosenblum and colleagues was used and modified by adding belt distance (BD) to the equation to account for the backward movement of the trailing limb from its initial contact, until the initial contact of the leading limb [15]. This modification was done to compensate for the lack of kinematic data (Fig 2).

The algorithm used computes mean and standard deviation (SD) of step length and step width by using a moving window of 6 samples to generate two graphs representing mean and SD of every step [15]. To analyze recovery performance after perturbations, 20 steps prior to the perturbation were defined as baseline walking. With the help of the algorithm an overall sum of deviation (OSDev) is computed by scanning mean and SD graphs [15]. OSDev is used to determine the number of steps used to recover gait. For this purpose, the algorithm searches for the exact point, in which the change in amplitude has become sufficiently small compared to pre perturbation as this indicates that the performance returned to baseline values [15].

In contrast to Rosenblum’s approach, we used ten instead of twenty samples, which was due to differences in the perturbation protocol. Whereas Rosenblum and colleagues used at least thirty-three seconds [15], our protocol contained twenty to thirty seconds between perturbations. As a twenty samples window would be too large for individuals with a low cadence, ten samples were used instead. To avoid errors in automatic step detection, the step, which triggered the perturbation, was excluded from analysis. As it was likely that some participants would not return their gait pattern to baseline levels, a maximum of fifteen steps after perturbation was set in line with previous research [31]. The procedure was used for step length, step width and step length and step width combined (total recovery).

### Statistical analysis

Participants’ baseline characteristics were analyzed using chi-square test for categorical variables, and independent t-test or, in case of non-parametric data, Mann-Whitney U test for continuous variables. Continuous variables were expressed as mean ± SD. Number of recovery steps were compared between the groups (OACH and OACI) using independent t-test. Between-group effect sizes were quantified using Cohen’s *d* with 0.2 representing a small effect, 0.5 a moderate sized effect, and 0.8 a large magnitude effect [32].

Given the exploratory nature of this sub-study, no a priori sample size calculation was performed. Instead, a convenience sample was analyzed in alignment with previous studies that have conducted similar investigations and included group sizes between eight to fifteen individuals [11, 15, 16].

Secondary analysis included a repeated measures of variance analysis (ANOVA). ANOVA was carried out with the within-subjects factor time (perturbation trial one and two) and the between subjects’ factor group (OACH and OACI) to explore differences between trials and groups. In case of significant time, group, or interaction effects, post hoc pairwise comparisons were conducted using Bonferroni correction for multiple pairwise comparisons. Due to the lack of an equivalent non-parametric analysis and due to ANOVA being relatively robust to violations of the normal distribution assumption [33] this analysis approach was used for all outcomes. Additionally, we investigated differences between perturbations by comparing the mean number of recovery of steps for both step length and step width averaged across groups and trials using the Kruskal-Wallis Test. In case of significant findings, we employed Bonferroni-corrected post hoc pairwise comparisons to determine differences between certain types of perturbations. Statistical significance was defined as *p* ≤ 0.05. Statistical analysis was conducted using SPSS for macOS (version 28.0; SPSS Inc., Chicago, IL, USA).

## Results

### Participant characteristics

For this exploratory sub-study full data sets of 36 participants were available, whose characteristics are reported in Table 1. Eighteen participants each were stratified into OACH and OACI group respectively. Besides cognitive function (p < 0.001), the proportion of males and females was different between the groups (p = 0.035). Further, the groups differed in treadmill walking speed with OACI walking more slowly than OACH (p = 0.012) but not in overground walking speed (p = 0.161).

**Table 1:** Characteristics of participants.

	OACI	OACH	p—value
**n**	18	18	
**Age, years (mean ± SD)**	74.7 ± 7.3	69.7 ± 7.8	0.064
**Sex (females/males)**	8/10	15/3	**0.035**
**BMI (mean ± SD)**	24.9 ± 3.8	26.9 ± 6.1	0.298
**MoCA (mean ± SD)**	21.9 ± 1.9	27.2 ± 1.6	**< 0.001**
**SPPB (mean ± SD)**	10.3 ± 1.4	10.8 ± 0.9	**0.318**
**Overground walking speed, km/h (mean ± SD)**	4.4 ± 0.6	4.6 ± 0.7	0.161
**Treadmill walking speed, km/h (mean ± SD)**	3.3 ± 0.8	3.8 ± 0.8	**0.012**

OACI = older adults with probable cognitive impairment; OACH = older adults, cognitively healthy; SD = standard deviation, MoCA = Montreal Cognitive Assessment, SPPB = Short Physical Performance Battery

### Recovery performance

**Fig 3:**
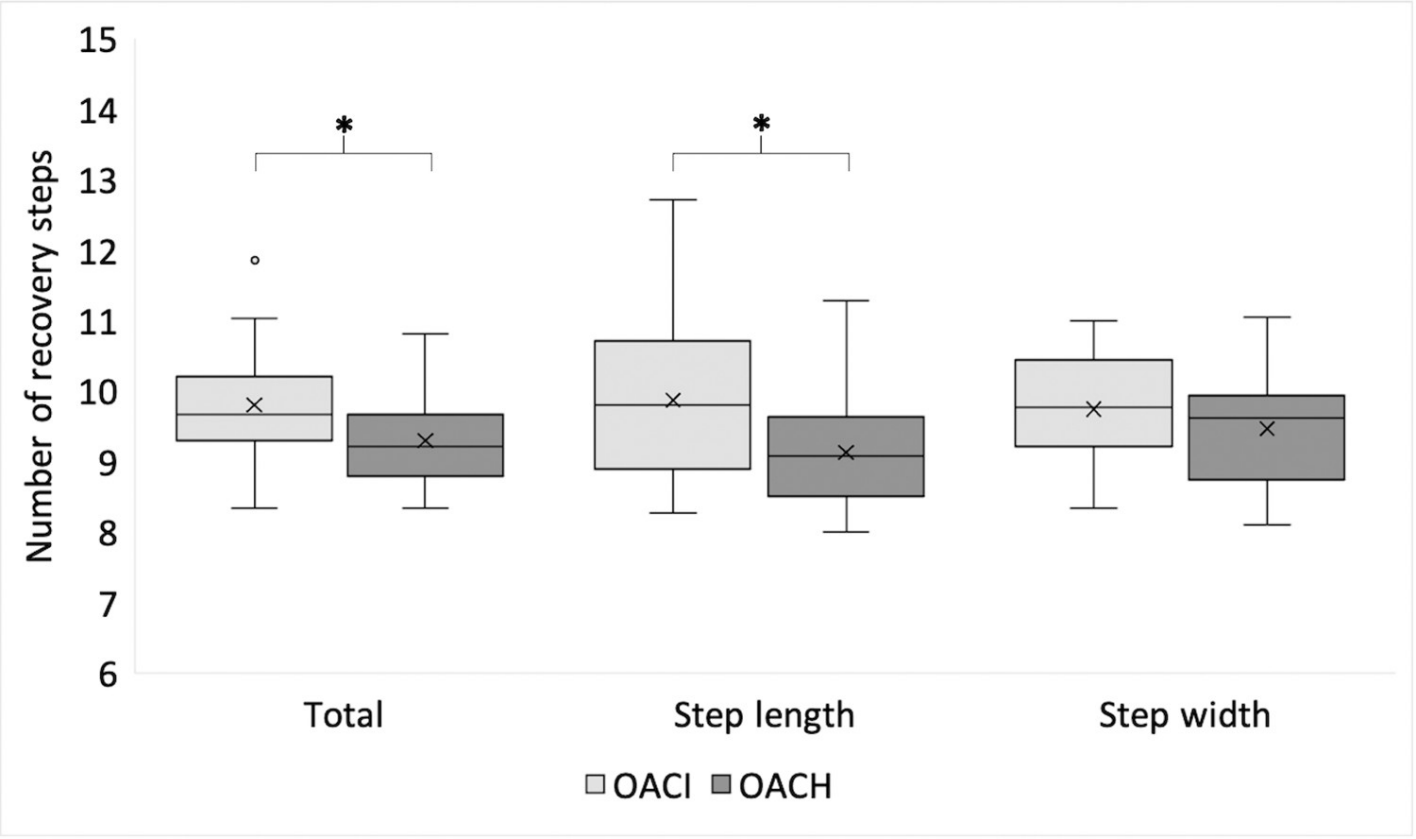
Boxplots of numbers of steps needed to return gait parameters to pre perturbation baseline (OACI = older adults with probable cognitive impairment; OACH = older adults, cognitively healthy; * = indicates significant difference (p < .05); for better visualization the y-axis starts at 6 instead of 0).

Fig 3 shows the recovery performance of the two groups for total recovery steps as well as step length and step width across all nine perturbations. Whereas the number of recovery steps for step width did not differ between OACH (mean: 9.5, SD: 0.8) and OACI (mean: 9.7, SD: 0.8; p = 0.151), the number of total recovery steps (p = 0.045, Cohen’s *d* = 0.69) as well as the recovery of step length (p = 0.039, Cohen’s *d* = 0.72) were significantly different between the groups. OACI (total number of steps: mean: 9.8, SD: 0.9; step length: mean: 9.9, SD: 1.2) needed significantly more steps to return to their baseline than OACH (total number of steps: mean: 9.3, SD:0.6; step length: mean: 9.1, SD: 0.8).

Table 2 presents a detailed breakdown of step length and step width recovery performance, categorized by perturbation type and trial. Secondary exploratory analysis unveiled significant time (p < 0.001) and between-group effects (p = 0.014) for step length recovery in the case of a trip to the right. While the overall recovery performance improved similarly in both groups between the first and second trials, individuals in the OACI group required significantly more steps than those in the OACH group to recover step length after a slip to their right foot. For step width, time effects were observed for trip right (p = 0.034), pitch up (p < 0.001), and full stop (p = 0.009), indicating a reduction in the number of recovery steps between the first and second trials across groups.

**Table 2:** Number of recovery steps sorted by type of perturbation and trial.

	OACI				OACH				p–value		
									Time	Group	Time*Group
Step length (number of recovery steps)											
	**P1**		**P2**		**P1**		**P2**				
**Slip right**	8.7	± 3.5	9.4	± 2.9	7.8	± 2.5	7.5	± 2.8	0.719	0.052	0.450
**Slip left**	8.2	± 3.4	8.3	± 2.9	8.2	± 2.4	7.8	± 3.2	0.796	0.651	0.740
**Trip right**	11.9	± 3.3	9.1	± 3.0	9	± 3.4	7.5	± 2.4	**< 0.001**	**0.014**	0.220
**Trip left**	11.3	± 3.7	10.1	± 3.1	10.3	± 3.3	9.7	± 3.5	0.152	0.480	0.608
**Sway left**	10.7	± 3.7	9.7	± 2.2	9.6	± 3.2	10.4	± 2.0	0.895	0.787	0.131
**Sway right**	10.9	± 3.4	10.6	± 2.3	10.3	± 3.6	8.9	± 2.7	0.185	0.887	0.847
**Pitch up**	10.8	± 3.2	9.2	± 2.6	9.7	± 2.9	9.8	± 2.7	0.297	0.234	0652
**Pitch down**	9.5	± 3.5	9.5	± 2.8	9.3	± 2.7	8.8	± 2.3	0.738	0.515	0.738
**Full stop**	10.4	± 3.8	9.4	± 2.7	10.5	± 3.7	9.3	± 3.5	0.114	0.976	0.906
**Step width** (number of recovery steps**)**											
	**P1**		**P2**		**P1**		**P2**				
**Slip right**	9.6	± 3.5	8.3	± 3.8	8.9	± 3.9	8.7	± 2.8	0.303	0.881	0.467
**Slip left**	9.9	± 3.9	7.8	± 3.2	7.8	± 3.3	8.4	± 3.2	0.308	0.085	0.412
**Trip right**	11.2	± 4.4	9.2	± 3.5	9.5	± 4.5	7.6	± 2.5	**0.034**	0.089	0.975
**Trip left**	10.8	± 4.1	8.6	± 3.3	9.7	± 3.4	8.7	± 2.8	0.056	0.543	0.500
**Sway left**	9.3	± 3.6	9.7	± 3.4	10.4	± 3.5	9.8	± 2.8	0.882	0.465	0.506
**Sway right**	9.7	± 4.1	9.7	± 3.3	8.5	± 3.0	8.8	± 3.4	0.185	0.887	0.847
**Pitch up**	13.2	± 2.0	9.2	± 2.8	11.5	± 3.5	9.9	± 2.5	**< 0.001**	0.522	0.054
**Pitch down**	10.7	± 4.0	10.7	± 2.2	11.1	± 3.7	9.7	± 2.5	0.402	0.704	0.402
**Full stop**	11.0	± 4.2	8.2	± 2.3	11.3	± 4.3	8.8	± 3.6	**0.009**	0.576	0.860

Values represent mean ± standard deviation; OACI = older adults with probable cognitive impairment; OACH = older adults, cognitively healthy; SD = standard deviation; P1 = perturbation trial one; P2 = perturbation trial 2; sway: left and right indicate the direction, in which the treadmill moved.

S1 Table provides an overview of the average number of recovery steps required per perturbation averaged across groups and trials. Kruskal-Wallis Test revealed a significant difference for both step width (p<0.001) and step length (p<0.001). While step length differed between 8.1 steps (slip left) to 10.4 steps (trip left), step width differed between 8.5 steps for slip left and 11.0 steps for pitch up. Significant differences were evident among various perturbations as demonstrated in S1 Table.

## Discussion

In this study, we disturbed gait in older adults, who had recently experienced a severe fall, walking on a perturbation treadmill with nine different types of perturbations. The participants represent a high-risk group for falls and functional decline as they were recruited after presenting to the ED after a fall. Our results support the hypothesis that OACI have a worse gait adaptability in comparison to OACH. Whereas recovery of step width did not differ between the groups, OACI needed more steps to return their step length back to pre-perturbation level than OACH.

Previous research indicated that single perturbations such as slips or trips do not cover the broad variety of perturbations that occur in everyday life [11, 28, 34, 35]. Therefore, the adequate adaptation of gait to different perturbations used in our study may present a more promising outcome to quantify a person’s reactive dynamic balance. Nonetheless, future studies are necessary to validate this approach, comparing the utilization of single or multiple types of perturbations and examining the potential association between individuals’ performance and future falls. This verification should encompass a prospective fall assessment. Moreover, future research should focus on investigating different types of perturbations to determine if certain ones pose greater challenges for individuals than others. This exploration could offer insights into specific training needs. Since perturbations were presented in a similar sequence, the observed recovery steps in our study may have been influenced by the order of their presentation, thereby limiting the generalizability of our results.

In our study, OACI had a poorer gait adaptability to balance perturbations, which was shown by the increased number of steps needed to return step length to baseline, in comparison to OACH. A poorer gait adaptability to balance perturbations in OACI is in line with previous research indicating differences in walking behavior between OACH and OACI [36–38]. As adapting step length is an important recovery mechanism to external perturbations [26, 39], immediate changes of an individual’s step length to perturbations were expected. However, the increased number of recovery steps needed by OACI may indicate less effective stepping responses, especially as Debelle and colleagues argue that a close to normal step length after a perturbation may present an important component of balance recovery [31]. However, previous results are controversial whether a close to normal, a shortened first step length (i.e., higher walking stability) or an elongated first step (i.e., better compensation for shifts in center of mass) may present better reactions to perturbations [26, 31, 39, 40]. Therefore, future research needs to target recovery mechanisms to different types and intensities of perturbations, to identify adequate recovery mechanisms and, thus, modify fall prevention efforts. According to the findings of our exploratory study, altering step length could be incorporated into falls prevention strategies to enhance gait adaptations, particularly among individuals with cognitive impairment.

The increased number of recovery steps in OACI in comparison to OACH may be due to slower cognitive processing and less cognitive flexibility, which is supported by the similarity in functional performance, as assessed with the short physical performance battery, across both groups. Impaired cognitive processing and less cognitive flexibility affect the ability to adapt behavior to external disturbances according to a previous review covering gait, cognition, and fall risks in older adults [41], which may explain findings in our study. In this context, our secondary, exploratory analysis appears promising as the observed time effects between trials one and two potentially indicate a trainability of gait adaptability to balance perturbations in both OACH and OACI. This warrants further investigation. As falls prevention interventions are not as effective in persons with cognitive impairment or dementia, although risk factors such as muscular strength can be adequately modified [42], dynamic reactive balance control might be an important target for falls prevention especially in these high-risk groups.

### Strengths and limitations

The decision to exclusively use data from force plates was motivated by clinical practice due to the approach being less time and cost consuming. Furthermore, force plates are well-established to measure ground reaction forces and, thus, provide kinetic information during walking [43]. While force plates offer an efficient and straightforward way of data collection, they lack the ability to provide accurate kinematic information. Step length, being a key kinematic parameter, is not directly measured by force plates. As a result, the insights gained from this method may not be as detailed and accurate as those obtained from marker-based systems. Thus, the local maximum of the CoP in anterior-posterior direction was defined as initial contact as it was more robust in regard to an automatic detection of steps. Nevertheless, it is likely that a minor time deviation to the true initial contact occurred. As this deviation has been similarly for all participants and across all step detection measures, it is unlikely to have affected results of the present study.

Due to not using a marker-based system, participants had to keep walking after the perturbations. To better reflect real life scenarios, self-paced walking approaches, which automatically and continuously adjust speed in real-time to an individual’s walking speed, may be used in future research studies.

Sample size was in line with previous studies. However, longitudinal approaches and bigger samples are warranted to analyze the relationship between recovery steps and future falls in individuals with and without cognitive impairment. In such studies sex differences in gait adaptability to perturbations may be further analyzed, as differences in group distribution could not be looked at in our study. Large scale studies would further benefit from an extensive neuropsychological test battery to confirm a formal diagnosis of mild cognitive impairment as well as an a priori sample size calculation.

## Conclusion

This study found moderate differences in gait adaptability to different perturbations in individuals with probable cognitive impairment when compared to cognitively healthy older adults using data from treadmill integrated force plates. Adaptation of step length was particularly challenging for individuals with probable cognitive impairment, which might be a link to providing effective falls prevention also for this group. As all participants had experienced an injurious fall about four weeks before their gait analysis, the approach used in this study may be valid to determine reactive balance performance in frail populations as well as in clinical practice. However, long-term observation and prospective fall assessments, as well as intervention trials are needed to support this notion.

## Supporting information

S1 Table Overview of the average number of recovery steps required per perturbation averaged across groups and trials. (DOCX)
