# Supplementary material for: Reduced adaptability to balance perturbations in older adults with probable cognitive impairment after a severe fall
Source: PLoS One. 2024 Jul 10;19(7):e0305067. doi: 10.1371/journal.pone.0305067 (PMC11236103; doi:10.1371/journal.pone.0305067)
Supplement: S1 Table — (DOCX) [file pone.0305067.s001.docx]

Supplementary Table1: Overview of the average number of recovery steps required per perturbation averaged across groups and trials.

|  | **Step length** | **Post hoc Test** | | **Step width** | **Post hoc Test** | |
| --- | --- | --- | --- | --- | --- | --- |
| **Slip right** | 8.4 ± 3.0 | Slip left | 0.649 | 8.9 ± 3.5 | Slip left  Trip right  Trip left  Sway left  Sway right  Pitch up  Pitch down  Full stop | 0.504 |
|  |  | Trip right | 0.057 |  |  | 0.330 |
|  |  | Trip left | <0.001 |  |  | 0.311 |
|  |  | Sway left | <0.001 |  |  | 0.114 |
|  |  | Sway right | <0.001 |  |  | 0.687 |
|  |  | Pitch up | 0.002 |  |  | <0.001 |
|  |  | Pitch down | 0.041 |  |  | 0.006 |
|  |  | Full stop | 0.004 |  |  | 0.098 |
| **Slip left** | 8.1 ± 3.0 | Trip right | 0.018 | 8.5 ± 3.5 | Trip right  Trip left  Sway left  Sway right  Pitch up  Pitch down  Full stop | 0.101 |
|  |  | Trip left | <0.001 |  |  | 0.093 |
|  |  | Sway left | <0.001 |  |  | 0.025 |
|  |  | Sway right | <0.001 |  |  | 0.284 |
|  |  | Pitch up | <0.001 |  |  | <0.001 |
|  |  | Pitch down | 0.012 |  |  | <0.001 |
|  |  | Full stop | <0.001 |  |  | 0.020 |
| **Trip right** | 9.4 ± 3.4 | Trip left | 0.060 | 9.4 ± 4.0 | Trip left  Sway left  Sway right  Pitch up  Pitch down  Full stop | 0.969 |
|  |  | Sway left | 0.061 |  |  | 0.545 |
|  |  | Sway right | 0.072 |  |  | 0.567 |
|  |  | Pitch up | 0.241 |  |  | 0.011 |
|  |  | Pitch down | 0.886 |  |  | 0.072 |
|  |  | Full stop | 0.324 |  |  | 0.495 |
| **Trip left** | 10.4 ± 3.4 | Sway left | 0.992 | 9.4 ± 3.5 | Sway left  Sway right  Pitch up  Pitch down  Full stop | 0.571 |
|  |  | Sway right | 0.934 |  |  | 0.541 |
|  |  | Pitch up | 0.477 |  |  | 0.012 |
|  |  | Pitch down | 0.082 |  |  | 0.078 |
|  |  | Full stop | 0.370 |  |  | 0.520 |
| **Sway left** | 10.1 ± 2.8 | Sway right | 0.942 | 9.8 ± 3.3 | Sway right  Pitch up  Pitch down  Full stop | 0.239 |
|  |  | Pitch up | 0.483 |  |  | 0.052 |
|  |  | Pitch down | 0.084 |  |  | 0.233 |
|  |  | Full stop | 0.375 |  |  | 0.939 |
| **Sway right** | 10.2 ± 3.0 | Pitch up | 0.530 | 9.2 ± 3.4 | Pitch up  Pitch down  Full stop | 0.002 |
|  |  | Pitch down | 0.098 |  |  | 0.018 |
|  |  | Full stop | 0.416 |  |  | 0.210 |
| **Pitch up** | 9.9 ± 2.9 | Pitch down | 0.304 | 11.0 ± 3.1 | Pitch down  Full stop | 0.451 |
|  |  | Full stop | 0.853 |  |  | 0.061 |
| **Pitch down** | 9.3 ± 2.8 | Full stop | 0.400 | 10.5 ± 3.2 | Full stop | 0.264 |
| **Full stop** | 9.9 ± 3.4 |  | | 9.8 ± 3.8 |  |  |
